# Application of Density Measurements for Discrimination and Evaluation of Chemical Composition of Different Types of Mechanically Separated Meat (MSM)

**DOI:** 10.3390/molecules27217600

**Published:** 2022-11-05

**Authors:** Piotr Kiełczyński, Piotr Szymański, Marek Szalewski, Krzysztof Wieja, Andrzej Balcerzak, Stanisław Ptasznik

**Affiliations:** 1 Institute of Fundamental Technological Research, Polish Academy of Sciences, ul. Pawińskiego 5B, 02-106 Warsaw, Poland; 2 Department of Meat and Fat Technology, Wacław Dąbrowski Institute of Agricultural and Food Biotechnology, 36 Rakowiecka Str., 02-532 Warsaw, Poland

**Keywords:** meat density, Mechanically Separated Meat (MSM), protein content, fat content, Sodium (*Na*) content, Calcium content (*Ca*)

## Abstract

At present, the problem of identifying and controlling different types of Mechanically Separated Meat (MSM) is a very important practical issue in the meat industry. To address this challenge, the authors propose a new, analytical method for the discrimination and characterization of MSM that uses density measurements. The method proposed by the authors, in contrast to the analytical methods existing so far, is rapid, non-destructive, relatively simple and can be computerized. The density measurements of meat samples were conducted with a modified pycnometric method. Statistically significant (p<0.01) differences were found in the evaluated mean values of density for all investigated types of meat. Subsequently, the density measurements were correlated with the physicochemical properties of meat samples. A high correlation coefficient was found between the density of meat samples and the content of protein, sodium and fat. The authors have proven that density measurements allow for rapid discrimination of various types of MSM, and can also be effectively used to determine the chemical composition of MSM samples, e.g., the content of protein, fat and sodium.

## 1. Introduction 

Mechanically Separated Meat (MSM), due to its low cost and high nutritional properties, is widely used in the food industry as a major ingredient in many processed meat products. Consequently, a particularly current and important problem is the characterization and investigation of the physicochemical properties of MSM. To this end, analytical tools are required that enable: (1) identifying different types of MSM and (2) evaluating the chemical composition of MSM. 

The need for these meat quality investigations and the necessity to specify the composition of the meat result from the recommendations of the European Union Regulations [1,2]. Pursuant to Regulation [1], MSM does not meet the definition of meat. According to Regulation [2], the composition of meat products must be clearly labeled. Therefore, it is an all the more pressing problem to develop rapid analytical methods that make it possible to investigate and identify the properties of different types of MSM as well as evaluate the presence of MSM in other meat products. Nowadays, due to the limitations of conventional methods, the development of new methods to characterize and investigate the physicochemical parameters of MSM is in accordance with the recommendations of the European Food Safety Authority [3]. 

To address this challenge, the authors propose a new rapid analytical method to discriminate various types of MSM, one that is based on the density measurements of the investigated MSM samples. To the authors’ knowledge, density measurements have not been yet used for identification of various types of MSM. 

Until now, the following methods have been used for MSM quality investigation and identification: (1) microscopic analysis [4], (2) X-ray fluorescence reflectometry [5], (3) micro CT [6,7], (4) electron spin resonance spectroscopy [8], (5) evaluation of radiostrontium levels [9], (6) ion chromatography with conductivity detection [10], (7) metabolite analysis [11], (8) biophysical methods [12], (9) hyperspectral imaging [13] and (10) near Infra-Red reflectance spectroscopy [14]. 

However, all these methods have numerous disadvantages [15], such as: (1) voluminous and high cost equipment, (2) prolonged and laborious measurements, (3) incapability to work in situ at production line, (4) very complex and time-consuming pre- and post-processing of large volume of data and (5) tedious and complicated calibration processes and procedures. 

For this reason, there is still a need to develop a new and rapid method for the identification of different types of MSM [16,17,18]. 

In order to overcome the disadvantages of existing methods, the authors propose the use of a new analytical method that is free from these drawbacks. This new method is based on measuring the density of the meat being investigated. The considered method is relatively simple and rapid, so it could also be ultimately used on the production line. The proposed method, apart from the ability to discriminate between different types of MSM, has an additional advantage, i.e., the analytical method proposed by the authors also enables the estimation of the chemical composition of meat (MSM). 

The main goal of the present work is to demonstrate the possibility of using density measurements to discriminate between different types of MSM, and additionally to find possible correlations between the density and chemical composition (constituents) of various kinds of MSM obtained using industrial methods. 

It worth noticing that, recently some attempts have been undertaken to explore density measurements to investigate the properties of meat materials [19,20,21,22,23]. However, these works are mainly focused on the investigation of the physicochemical properties and chemical composition of meat materials, not on the discrimination of various types of MSM. 

However, the methods proposed in [19,20,21,22,23] exhibit also some disadvantages. For example, in [23], meat samples were fabricated artificially with a specified fat content, the density of which was then evaluated. This is a labor-intensive and time consuming method. 

By contrast, in the study presented by the authors, we investigated meat samples obtained in actual technological processes on the production line at the meat factory. For the first time, the densities of the same type of meat (chicken) obtained using different methods were compared and identified. The method proposed by the authors makes it possible to identify chicken meat obtained in various fabrication processes (e.g., manually deboned meat and mechanically separated meat). This is a novelty. On the other hand, contrary to our research, the investigations conducted in [19,20,21,22,23] did not identify different types of meat. 

Compared to conventional methods, the authors’ method uses a simple research methodology and analytical tools (based on meat density measurements) that allow the obtaining of important technological characteristics of meat (i.e., the discrimination of various types of meat, such as hand deboned and MSM) which are critical in the meat industry. 

## 2. Materials and Methods 

### 2.1. Samples Preparation 

The raw material for investigations was the chicken (*Gallus gallus domesticus*) meat supplied by a meat factory in Poland. Accordingly, the raw material was the chicken breast meat obtained from the industrial manual deboning (MD) of poultry meat and four types of mechanically separated meat obtained from mechanical deboning of non-frozen chicken carcasses and chicken collarbones using two different variants of separator devices, namely: (1) low pressure device—Sepamatic Sepa 1200 belt separator device (Overath, Germany) with holes in the stainless steel drum with a diameter of 3.0 mm. In this device, the tissue is passed between a rubber belt and a micro-grooved steel drum. The meat passes through the holes while bones, skin and thicker layers of connective tissue remain outside of the drum and are thrown out through a discharge chute. Due to the design of the device, the bones for de-boning were directed after the initial fragmentation; (2) high pressure device—Lima RM 600 s separator (Quimper, France) operating at a pressure of 1.5 MPa. The size of the raw material outlet (soft parts) gaps is 0.5 mm × 20.0 mm. In this case, the ground bone and meat mixture is introduced into a screw-driven boning head. The material is pressed (with increasing pressure), and the meat is squeezed through a perforated steel cylinder encasing the auger [3]. Bone and connective tissue particles that cannot pass through the perforated cylinder are pushed forward and exit at the end of the head. The bones for deboning were directed without any initial fragmentation. 

Prior to investigation, the MD chicken meat and MSM samples were ground with an Edesa PL-22-TU-T device (Czosnów, Poland) and homogenized with a Keripar mixer (Troy, OH, USA) for 5 min. The temperature of the raw material after mixing was between 6 and 7 °C. 

In this study, we investigated five types of chicken meat samples provided by different technological processes: (1) minced MD chicken fillets, (2) low pressure MSM samples extracted from carcasses, (3) low pressure MSM samples extracted from collarbones, (4) high pressure MSM samples extracted from carcasses and (5) high pressure MSM samples extracted from collarbones. 

### 2.2. Density Measurements 

To measure the density ρ (in g/cm^3^) we adapted a pycnometric method described briefly below. The weight of meat samples, empty test flask, test flask with water, etc. was measured with an analytical balance (A 120S, Sartorius, Goettingen, Germany) with an accuracy 0.1 mg. A test flask with a volume of 250 mL was used. The mass of each selected sample was chosen approximately as 20 g. 

At the beginning, we measure the sample mass m. Next, the volumetric cylinder was filled with water having a volume of about 100 cm^3^. Subsequently, the investigated meat sample was inserted into a measuring cylinder partially filled with water (~100 cm^3^). Finally, the volume V of the meat sample was determined from the changes of the water level. 

Knowing the mass m and volume V of the measured sample, the density of the meat sample was determined from the standard formula $\rho =m/V\;\left[g/{\mathrm{cm}}^{3}\right]$. The expanded 2σ relative uncertainty for the density measurements was estimated according to the ISO guidelines [24]. Namely, the expanded 2σ relative uncertainty for the density Δρ/ρ can be expressed as:

(1) $$\frac{\Delta \rho }{\rho }=2\sqrt{{\left(\frac{\Delta m}{m}\right)}^{2}+2{\left(\frac{\Delta L}{L}\right)}^{2}}$$

where: $\frac{\Delta m}{m}=10^{-5}$ is the relative standard uncertainty of the mass measurements and $\frac{\Delta L}{L}=10^{-3}$ is the relative standard uncertainty of the water level measurements. As a result, the expanded relative uncertainty of the density measurements equals $\pm 3\cdot 10^{-3}\;g/{\mathrm{cm}}^{3}$. Density measurements were carried out at an ambient temperature of 24 °C. 

### 2.3. Chemical Parameters of the Investigated Meat Samples 

The chemical composition of the investigated meat samples was determined using standard analytical methods. Chemical analysis of MSM samples involved the determination of:

(a) Protein content—the protein amount [%] was measured employing a Kjeldahl method (Foss Tecator, Hoeganaes Sweden), according to the ISO standard [ISO 937:1978]. The method determines at first the total amount of N (nitrogen). The temperature of mineralization was 420 °C. Subsequently the total amount of nitrogen (N) is converted to the amount of protein using a conversion factor (for meat = 6.25).
(b) Fat content—the fat was extracted with a Soxhlet technique (Tecator Co., Sweden). The amount of fat (in %) was next determined by a weight method according to the ISO Standard [ISO 1444–1996 (R2018)]. The drying process was performed at a temperature of 103 °C.
(c) Sodium content—the amount of sodium Na (in mg/kg) was determined by employing flame atomic absorption spectrometry (FAAS) working in conjunction with the Hitachi Z-2000 apparatus, Japan. The temperature of mineralization was 420 °C.
(d) Phosphorus content—the amount of the total phosphorus P content (%), expressed as $P_{2}O_{5}$, was estimated according to the Polish Standard [PN-A-82060:1999] using the following steps: (1) sample mineralization, at a temperature of 560 °C, (2) extraction of P in the form of choline phosphoromolybdate and (3) measurement of weight in order to determine the total amount of P.
(e) Calcium content—the amount of calcium Ca (in mg/kg) was determined by employing flame atomic absorption spectrometry (FAAS) working in conjunction with the Hitachi Z-2000 apparatus, Japan. The measuring process can be divided into the following steps: (1) mineralization of the meat sample at a temperature of 420 °C, (2) dissolution, (3) addition of a matrix modifier, lanthanum buffer and (4) evaluation of Ca content on a flame spectrometer using a Ca lamp (wavelength = 422.7 nm).
(f) Water content—the measured meat samples were subjected to a 30-min drying process at a temperature of 103 °C (Oven Series 9000, Thermolyne, Waltham, MA, USA). The amount of H_2_O was evaluated employing a conventional drying method according to the ISO Standard [ISO 1442: 1997 (R2018)].

A detailed description of each of the methods used to determine the content of: (a) protein, (b) fat, (c) water, (d) phosphorus, (e) calcium and (f) sodium is included in the Supplementary Materials. 

### 2.4. Statistical Analysis 

#### 2.4.1. Method of Sample Preparation 

The samples for the investigation were selected in a way that ensured high randomization of the sample selection. Representative meat samples for analysis were obtained as follows. From meat factory, we received raw material of meat from 3 different randomly selected production batches. From each production batch we obtained 5 packages (5 kg each) of 5 different types of meat, namely: (1) hand deboned chicken fillets, (2) low pressure MSM from chicken carcasses, (3) low pressure MSM from chicken collarbones, (4) high pressure MSM from chicken carcasses and (5) high pressure MSM from chicken collarbones. In total we obtained 5 × 3 = 15 packages of different types of meat (5 kg each). Subsequently, all 3 packages of 5 different types of meat were minced and mixed. As a result, we obtained 5 packages (15 kg each) of 5 different kinds of meat. 

During the investigation of 5 different types of MSM (high pressure, low pressure, etc.), only one separate sample was randomly selected for each individual measurement. For example, for measuring the density of MSM of the high-pressure carcass type, the number of samples taken was 5. As 5 different types of meat were measured, the number of independent randomly extracted meat samples to measure the density of all types of meat was 5 × 5 = 25. Since the measurements of the density and 6 other physicochemical parameters of MSM (protein, fat, sodium (Na), calcium (Ca), phosphorus (P) and water ($H_{2}O$) content) were performed 5 times, the total number of independent samples was (1 + 6) × 5 × 5 = 175. Consequently, 175 samples were randomly selected for measurement. Each sample was measured only once. 

#### 2.4.2. Statistical Procedures 

Statistical analysis of the results was conducted using the Statistica 13.3 software (Tibco Software Inc., Palo Alto, Santa Clara, CA, USA). To assess the differences in density of various kinds of investigated MSM, a one-way ANOVA procedure was used. Post-hoc Tukey’s tests were performed to prove that the mean values of the density of different types of meat differ significantly at p<0.01. The correlations between the density and the amount of protein, fat, Na, P, Ca and $H_{2}O$ were determined. 

The linear regression curves of the measured density versus content of: protein, fat, Na, P, Ca and $H_{2}O$ were evaluated. Additionally, we evaluated the correlation matrix that shows the Pearson correlation coefficients r between all 7 physicochemical parameters of investigated MSM samples. 

## 3. Results 

### 3.1. Density Measurements 

**Figure 1:**
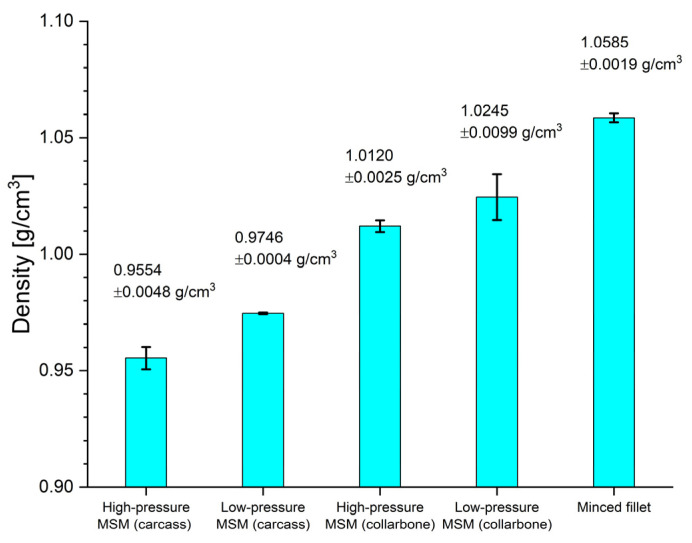
Graph of the mean values of density ρ for different types of MSM with the corresponding confidence intervals: mean value ± standard deviation.

Density measurements were carried out using five (5) independent randomly selected samples of each the five (5) type of investigated MSM (in total 5 × 5 = 25 independent meat samples were used). The results of the density measurements for all 25 meat samples performed by the modified pycnometric method are presented in Figure 1. 

Significant differences between the average density of different types of meat were assessed in the following analytical steps: (1) normal distribution check (Shapiro-Wilk test), (2) homogeneity of variances analysis (Levene’s test) and (3) post-hoc Tukey tests and one-way ANOVA procedure. According to this analysis, the mean values of the density ρ of the investigated various types of meat samples are statistically different (p<0.01). 

The results of the density ρ measurements, mean values and standard deviations for the investigated various types of meat samples are represented graphically in Figure 1. 

The statistical analysis shows that the mean values of the density ρ for the investigated samples of various types of MSM are significantly different (p<0.01). This observation confirms our assertion that the density measurements can be effectively used in the identification of different types of MSM. 

### 3.2. Physicochemical Parameters of Meat Samples 

In order to determine the correlation between the density and chemical composition of the investigated MSM samples, using standard analytical methods (see Section 2.3), the content of: (1) protein, (2) fat, (3) Na, (4) Ca, (5) P and (6) $H_{2}O$ were evaluated and summarized; see Table 1. The main factor influencing the chemical composition of MSM is the technique of its manufacturing. 

Five (5) randomly selected samples of each five (5) sorts of chicken meat, i.e., manually deboned (MD) meat, two (2) types of low-pressure MSM, and two (2) types of high-pressure MSM (in total 5 × 5 × 6 = 150 independent meat samples) were measured to characterize the chemical composition (6 parameters) of the investigated meat samples. 

### 3.3. Correlations between Density and Chemical Composition of MSM Samples 

The obtained results were statistically analyzed using the Statistica 13.3 computer program, determining the correlation coefficients between the density and individual chemical components (constituents) of the MSM samples. We have determined the following statistical parameters: Pearson’s correlation coefficients r, p values and linear regression equations. 

#### 3.3.1. Correlation Matrix 

We performed the cross-correlation analysis (Pearson’s correlation coefficients r) between all chemical parameters of meat samples and their density. Consequently, we obtained the following cross-correlation matrix; see Table 2. 

The results of the statistical analysis reveal (see Table 2) that the density ρ is strongly positively correlated with the content of protein (r=0.9646). 

Highly significant negative correlations were determined between the density and the content of fat (r=−0.8827) and Na (r=−0.8752). This means that the density increases with increasing protein content and decreases as the fat and Na content increases. 

#### 3.3.2. Graphs of Linear Regression Curves 

We have determined the equations of the linear regression for individual correlations, i.e., for the correlations of density ρ with the amount of (1) protein, (2) fat, (3) Na, (4) $H_{2}O$, (5) P and (6) Ca; see Table 3. 

y = density, x = content of: protein, fat, sodium, calcium, phosphorus and water, respectively. 

**Figure 2:**
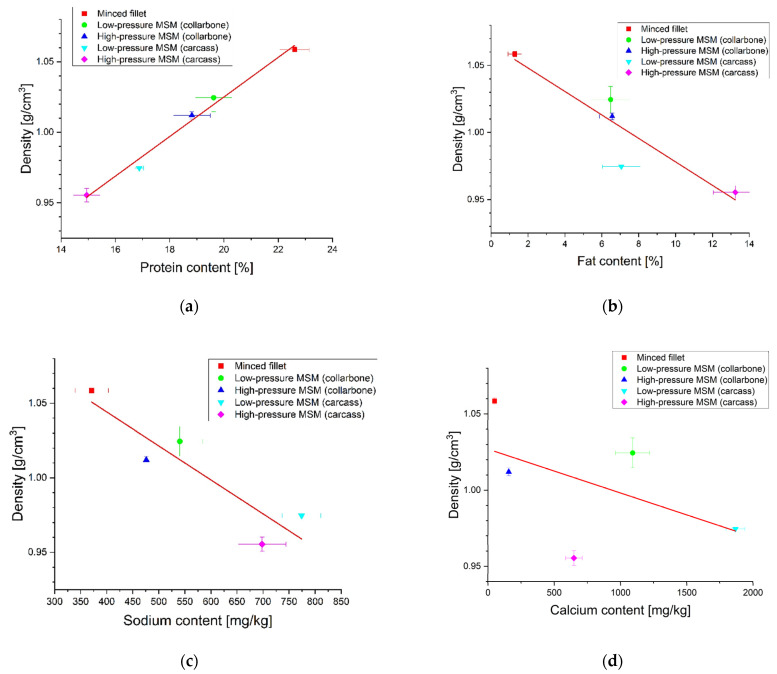

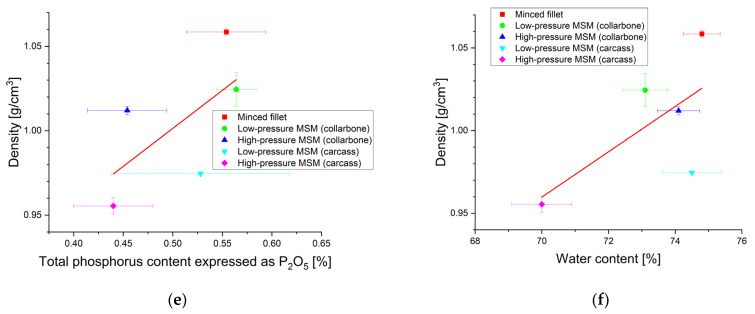
Linear regression curves relating measured density ρ with the content of (**a**) protein, (**b**) fat, (**c**) sodium, (**d**) calcium, (**e**) phosphorus and (**f**) water.

Exemplary regression curves that relate the density ρ with the content of: (a) protein, (b) fat, (c) Na, (d) Ca, (e) P and (f) $H_{2}O$ are portrayed in Figure 2a–f. 

From Table 3 it is evident that statistically significant linear regression equations were obtained for the following correlations: (a) density—protein content (p<0.00067), (b) density—fat content (p<0.0325), and (c) density—Na content (p<0.0304). 

## 4. Discussion 

The process of identifying various meat products in industrial conditions should be fast, cheap, non-destructive and carried out using compact, portable and fully automated equipment. The new analytical method proposed by the authors, based on density measurements, meets all these requirements. 

In general, conventional methods for investigation meat properties are very complex (e.g., Hyperspectral Imaging). These methods use sophisticated and very expensive equipment and require complicated and time-consuming preparation of samples in preprocessing and the elaboration of the results in post-processing. The key element of these methods is the use of complex statistical methods. These methods give results that are difficult to interpret and ambiguous. 

Contrary to these conventional methods, the analytical method proposed by the authors for evaluation of meat properties based on meat density measurements is relatively simple, inexpensive and does not require complicated preprocessing and post-processing data analysis algorithms. The density of the investigated samples is obtained by means of simple operational relationships given by analytical formulas. 

### 4.1. Variations of the Measured Density 

The measured density ρ of the investigated meat samples ranged from ρ=0.9554 $g/{\mathrm{cm}}^{3}$ (MSM samples obtained with high pressure separation of meat from bones of chicken carcasses) to ρ=1.0585 $g/{\mathrm{cm}}^{3}$ (meat samples obtained from HD chicken fillets). This indicates that meat samples obtained from chicken fillets are denser than MSM samples obtained by high pressure separation of meat from bones of chicken carcasses, which suffered destruction of muscle fiber structure during the process [25]. 

The difference Δρ=0.1031 $g/{\mathrm{cm}}^{3}$ between the maximum and minimum measured densities (for HP MSM—carcass and Minced fillet) is quite large (9.7%). This is an evident advantage of the proposed analytical method, because from the density measurements we are able to reliably deduce the type of the meat sample under investigation (see Figure 1 and Figure 2a–c). 

The difference in density ρ between two consecutive values of ρ for various types of meat (see Figure 1) is quite clear and ranges from 0.0125 $g/{\mathrm{cm}}^{3}$ to 0.0382 $g/{\mathrm{cm}}^{3}$. This suggests that the proposed analytical method that uses the measurement of the density ρ can be effectively applied to differentiate between different types of meat samples. 

### 4.2. Correlation between the Measured Density and the Content of Basic Chemical Components 

The main feature of the proposed analytical method, based on the density measurements, is high degree of correlation between the measured density ρ and the content of basic chemical components in the measured meat samples (see Figure 2a–c). 

#### 4.2.1. Protein Content 

The correlation coefficient r between the density ρ and the protein content in the investigated meat samples was significant (p=0.00067) and amounts to r=0.9646; see Table 3. The protein density is higher than the average meat density. Therefore, a higher protein content increases the overall density of the investigated meat samples, see Figure 2a. 

#### 4.2.2. Fat Content 

A highly significant (p=0.0325) correlation coefficient between the density ρ and the fat content (r=−0.8827) was also found, see Table 3. This can be explained by the fact that fat has a lower density than the average density of meat. Hence, a higher fat content reduces the overall density of the investigated meat samples; see Figure 2b. 

#### 4.2.3. Sodium Content 

It is worth noting that highly significant correlations were also found between density ρ and Na content (r=−0.8752 and p=0.0304); see Table 3. Since the density of Na ($\rho_{Na}=0.97\;g/cm^{3}$) is generally lower than that of meat, the addition of pure Na to the meat will reduce the effective density of the investigated meat sample. Therefore, with increasing Na content, density ρ of meat samples should diminish, which is in accordance with Figure 2c. 

#### 4.2.4. Phosphorus and Water Content 

Due to the low variability of the P and $H_{2}O$ content in the investigated meat samples, it was difficult to find a significant linear correlation between the density ρ and the content of P and $H_{2}O$; see Table 3 and Figure 2e,f. 

#### 4.2.5. Calcium Content 

The experimental data (Table 1) show a large distribution of Ca content in the investigated meat samples. As expected, the lowest Ca content was found in samples of the HD chicken fillets. Surprisingly, the highest Ca content was found in samples obtained by low pressure methods, i.e., (a) low pressure MSM samples from chicken carcasses and (b) low pressure MSM samples from chicken collarbones. This was not the case in samples obtained by high pressure methods, i.e., (c) high pressure MSM samples from chicken carcasses and (d) high pressure MSM samples from chicken collarbones. 

The above statement can be explained by the fact that the applied low pressure methods with the appropriate setting of operational parameters may result in meat products with a relatively high bone content, greater than that resulting from the use of high pressure methods. In addition, the calcium content of MSM and HD meat samples varies depending on the species of animal, the part of the carcass and method (technical conditions) used to recover the meat. 

In several publications (e.g., in the EFSA recommendations [3]), the measurement of Ca content was proposed as a criterion for the presence of MSM in the investigated meat samples. However, the authors’ research shows the opposite conclusion. We obtained a fairly low correlation coefficient (r=−0.5289) between the density measurement and the Ca content. Therefore, our research demonstrates that measuring the Ca content cannot be used as a reliable criterion for the presence of MSM. 

The performed statistical analysis (see Section 2 and Section 3) shows that the mean values of the density ρ of different types of MSM are statistically different (p<0.01). Therefore, the analytical method developed by the authors, based on density ρ measurements, can be successfully used for discrimination of different types of MSM. The analytical method proposed in this paper is rapid and significantly relaxes the requirement for the involvement of highly qualified personnel, and can be recommended as a quick and effective analytical technique for the identification of different classes of meat, e.g., to distinguish hand deboned (HD) meat from mechanically separated meat. 

By contrast, conventional methods to characterize and discriminate different types of meat (including MSM), such as, e.g., (a) Near Infra-Red hyperspectral imaging method [26,27] and (b) Near Infra-Red Reflectance spectroscopy method [28], are very complex, requiring time-consuming pre-processing and post-processing stages to effectively recover useful information from the large amount of data obtained. For this reason, conventional methods can hardly be envisaged as candidates for implementation on the production line. 

As a bonus, the analytical method developed in the current study can be used to estimate the chemical composition of meat products. This is an important advantage of the developed method since the quality of meat products and the parameters of the technological processes are directly related to the chemical composition of the raw materials used. 

By contrast, the conventional analytical methods for determining the chemical composition of meat are quite burdensome and laborious, therefore they are unsuitable for on-line industrial applications. 

Since the analytical method proposed in this paper, which consists of measuring the density ρ of the examined meat products, is relatively simple and devoid of the deficiencies of the conventional methods, we anticipate that in future the proposed method can be used to analyze the quality, chemical composition and discrimination of other types of processed meat, such as pork, beef, fish, etc., on-line in industrial practice. 

The results obtained in this study are new and original. Literature reports indicate that so far density measurements of meat have focused mainly on modeling of thermal technological processes [29,30]. In contrast, our study aims to discriminate MSM from HB meat using density measurements. 

To the best of our knowledge, the analytical method presented in this paper, based on density measurements, has not yet been used to distinguish between different types of meat, e.g., to discriminate chicken fillets from mechanically separated chicken meat. 

All measurements were carried out in accordance with ISO standards, with the use of conventional physicochemical methods, validated and used in the accredited laboratory of the Institute of Agricultural and Food Biotechnology in Warsaw, Poland, that meets the norms of the PN-EN/ISO 17025: 2018 standard. As a result, the credibility of the obtained results was ensured. 

## 5. Conclusions 

From the research and analyses performed in this paper, the following main conclusions can be drawn:

1. The analytical method developed in this study, based on the measurements of the density ρ, can be efficiently applied to distinguish and characterize various types of MSM obtained with different technological methods.
2. Measurements of the density ρ can also be used to estimate the chemical composition of MSM samples such as the content of: protein, fat and Na, respectively.
3. High correlation coefficients between the density ρ and the content of protein (r=0.9646), fat (r=−0.8827), and Na (r=−0.8752), exist in the investigated MSM samples.
4. Moderate values of the correlation coefficient were recognized between the density ρ and the content of $H_{2}O$ (r=0.6173), Ca (r=−0.5289) and P (r=−0.4393).
5. Statistically significant linear regression curves relating the measured density ρ of the MSM samples and the content of: protein (p<0.00067), fat (p<0.0325) and Na (p<0.0304) were determined.
6. Manually deboned meat (filet), in relation to all types of MSM, is characterized by the highest density, the highest protein content and the lowest Na, Ca and fat content.

By contrast to the currently available conventional methods, the analytical method proposed in this paper for the identification and investigation of various types of MSM, based on the use of density ρ measurements, is rapid and relatively simple and has the potential for on-line implementations. 

The authors hope that the results of the research presented in this paper can be of interest for food processing engineers as well as for researchers working in Food Chemistry and modeling, design and optimization of technological processes in the Food Industry. However, the application of the analytical method proposed in this paper to estimate the falsification of the quality of various types of meat requires further research. The application of density measurements to differentiate MSM from other meat products requires additional investigations and will be the subject of future authors’ works. 

## Figures and Tables

**Table 1:** Measured chemical composition of the investigated meat samples. Mean values and standard deviations are given in square and round brackets respectively. Mean values in the same column equipped with different superscript letters A, B, C, and D are statistically different (p<0.05).

Type of Meat	Protein Content [%]	Fat Content [%]	Sodium Content [mg/kg]	Calcium Content [mg/kg]	Total Phosphorus Content Expressed as P_2_O_5_ [%]	Water Content [%]
Minced MD chicken fillet	23.1 22.9 22.3 21.8 22.9	0.9 1.0 1.5 1.8 1.2	378 326 411 356 385	48 52 45 48 55	0.56 0.53 0.50 0.58 0.60	75.4 75.3 74.1 74.5 74.6
	[22.6] ^A^ ± (0.5)	[1.3] ^A^ ± (0.4)	[371] ^A^ ± (32)	[50] ^A^ ± (4)	[0.55] ^A,B^ ± (0.04)	[74.8] ^A^ ± (0.6)
Low-pressure MSM (collarbone)	20.5 20.1 19.5 19.1 18.9	5.5 6.0 5.8 7.1 8.0	517 610 509 556 510	1280 960 1080 1150 990	0.59 0.55 0.54 0.56 0.58	73.5 73.2 73.8 73.1 72.0
	[19.6] ^B^ ± (0.7)	[6.5] ^B^ ± (1.0)	[540] ^B^ ± (43)	[1092] ^B^ ± (129)	[0.56] ^B^ ± (0.02)	[73.1] ^B^ ± (0.7)
High-pressure MSM (collarbone)	18.6 19.8 19.2 18.4 18.1	6.8 5.6 7.2 7.1 6.1	449 443 490 475 523	152 170 144 160 158	0.44 0.45 0.40 0.48 0.50	74.7 74.3 73.5 73.9 75.1
	[18.8] ^B^ ± (0.7)	[6.6] ^B^ ± (0.7)	[476] ^B^ ± (33)	[157] ^A^ ± (10)	[0.45] ^C^ ± (0.04)	[74.1] ^B,C^ ± (0.6)
Low-pressure MSM (carcass)	16.8 17.0 17.1 16.8 16.7	7.1 6.3 5.9 7.5 8.5	761 733 752 823 801	1840 1760 1910 1880 1947	0.59 0.42 0.65 0.45 0.53	75.1 74.6 75.4 74.3 73.1
	[16.9] ^C^ ± (0.16)	[7.1] ^B^ ± (1.0)	[774] ^C^ ± (37)	[1867] ^C^± (72)	[0.53] ^A,B,C^ ± (0.09)	[74.5] ^B,C^ ± (0.9)
High-pressure MSM (carcass)	15.0 14.2 15.5 15.2 14.8	14.8 14.0 12.0 13.2 12.2	635 679 723 698 756	720 560 620 692 650	0.45 0.42 0.45 0.50 0.38	68.8 69.4 70.1 69.0 71.0
	[15.0] ^D^ ± (0.5)	[13.2] ^C^ ± (1.2)	[698] ^D^ ± (46)	[648] ^D^ ± (63)	[0.44] ^C^ ± (0.04)	[70.0] ^C^ ± (0.9)

**Table 2:** Cross-correlation matrix (Pearson’s correlation coefficients r) for physicochemical parameters of the investigated MSM.

	Density	Meat Sort	Protein	Fat	Water	Sodium	Phosph.	Calcium
density	1.000000	0.983204	0.964552	−0.882701	0.617270	−0.875236	0.439317	−0.528908
meat sort	0.983204	1.000000	0.970714	−0.892136	0.624469	−0.834264	0.529698	−0.417043
protein	0.964552	0.970714	1.000000	−0.930942	0.664178	−0.844761	0.480469	−0.464711
fat	−0.882701	−0.892136	−0.930942	1.000000	−0.863492	0.680606	−0.514032	0.271081
water	0.617270	0.624469	0.664178	−0.863492	1.000000	−0.393946	0.412542	0.003036
sodium	−0.875236	−0.834264	−0.844761	0.680606	−0.393946	1.000000	−0.220277	0.791389
phosph.	0.439317	0.529698	0.480469	−0.514032	0.412542	−0.220277	1.000000	0.226052
calcium	−0.528908	−0.417043	−0.464711	0.271081	0.003036	0.791389	0.226052	1.000000

**Table 3:** Pearson’s coefficients r, p-values and linear regression equations for density ρ versus content of: protein, fat, (Na), (Ca), (P) and ($H_{2}O$).

Meat Property	Linear Regression Equation	p-Value	Pearson Correlation Coefficient *r*
**Protein content**	y = 0.0140x + 0.7443	0.00067	0.9646
**Fat content**	y = −0.0087x + 1.0656	0.0325	−0.8827
**Sodium content**	y = −0.00023x + 1.1353	0.0304	−0.8752
**Calcium content**	y = −0.000028x + 1.0269	0.3636	−0.5289
**Phosphorus content**	y = 0.45037x + 0.7763	0.2517	0.4393
**Water content**	y = 0.01370x + 0.00037	0.2230	0.6173

## Data Availability

Not applicable.

## Supplementary Materials

The following supporting information can be downloaded at: https://www.mdpi.com/article/10.3390/molecules27217600/s1.
