# Supplementary material for: Application of Density Measurements for Discrimination and Evaluation of Chemical Composition of Different Types of Mechanically Separated Meat (MSM)
Source: Molecules. 2022 Nov 5;27(21):7600. doi: 10.3390/molecules27217600 (PMC9654400; doi:10.3390/molecules27217600)
Supplement: Supplementary file 1 [file molecules-27-07600-s001.zip › molecules-1895513-supplementary.pdf]

Supplementary Materials

I. Description of methods for determining the chemical compositions of the investigated meat samples:

1) Phosphorus content

The method consists in mineralizing the sample, precipitating phosphorus in the form of choline phosphoromolybdate and determining the total phosphorus content by weight. 2.5 g of the sample was weighed to an accuracy of 0.001 g into a quartz crucible calcined to constant weight and cooled down in a desiccator. The sample was pre-ashed on a heating plate (Schott Ceran 500®, Robax, Germany) until no more black smoke was evolved. It was then burned in an incinerator (62700 Furnace, Barnstead Thermolyne, United States) at 560 ° C until an ash was uniformly white to off-white. After cooling, 25 ml of dilute nitric acid (V) was added to the ashed sample and heated for 30 min in a boiling bath. Then the content of the crucible were filtered through a filter paper (medium quality filters 150 mm) into a 400 ml beaker, rinsing the crucible and the filter with water.
In parallel, a solution for blank test was prepared by mixing 25 ml of dilute nitric acid (V) with 75 ml of water. 50 ml of precipitation reagent were added to the obtained solutions, the beakers were covered with cover slips and placed on a heating plate. Cooked for 1 minute from the moment of boiling. The samples were cooled to room temperature. By means of a glass rod, the precipitate was transferred to a Gooch funnel, previously dried to constant weight at 250 °C and fixed in a suction flask. The precipitate was washed 5 times with 25 ml of water each time. The funnel with the precipitate was dried at 250 °C until constant weight, and then, after cooling down in a desiccator, it was weighed on an analytical balance.

2) Calcium and sodium content

All solvents, reagents and calcium and sodium standards supplied by Merck (Darmstadt, Germany) were used in this work. Standard solutions of calcium and sodium were prepared from separate 1000 mg/l standards. Six working standards for

each element were set from the previous solutions. The concentration of calcium and sodium in the tested samples were obtained from calibration graphs.

The content of calcium and sodium was determined using the flame atomic absorption spectrometry (FAAS) technique. The test samples were prepared and mineralized according to own research procedure PS-01 3rd edition, 6th July 2009: "Determination of tin, manganese, chromium, sodium, potassium, calcium, magnesium by atomic absorption spectrometry in food products".

2 g of the samples were weighed into the quartz crucibles, ashed on a heating plate and dry mineralized in a muffle furnace at 420˚C. Concentrated nitric acid was added to the ash, heated and burned again at 420˚C in a muffle furnace to obtain a white ash. Ash was dissolved in 1 mol/l nitric acid and filled up in 10 ml volumetric flasks by the same acid.

In order to determine calcium, lanthanum chloride buffer was added to appropriately diluted samples in an amount such that the cesium concentration was the same in the standards and the tested sample. Hollow cathode lamp for Ca (PHOTRON PTY.LTD.) was used to determination.

In order to determine sodium, cesium chloride buffer was added to appropriately diluted samples in an amount such that the cesium concentration was the same in the standards and the tested sample. Hollow cathode lamp for Na (PHOTRON PTY.LTD.) was used to determination.

Each sample was analyzed in duplicate. The HITACHI Z-2000 atomic absorption spectrometer was used for the determination. All the conditions applied for calcium and sodium determinations are given in Table 1.

Table S1. Instrument settings.

Parameters	Ca	Na
Wavelength, nm	422.7	589.0
Lamp current, mA	7.5	10.0
Slit width, nm	1.30	0.40
Air flow rate, dm^3/h	400	400
Acetylene flow rate, dm^3/h	60	60

3) Protein content

Total protein content by the Kjeldahl method.

The method consists in determining the total nitrogen content by the Kjeldahl method and then using a factor (6.25 for meat) to calculate the protein content.

A sample of 1 g was weighed to an accuracy of 0.001 g and transferred to the distillation tube. Then 15 cm^3 of concentrated sulfuric acid (VI) and 2.5 g of catalyst were added. The contents were mineralized in a FOSS Kjeltec Digestor 2006 digestion block (Foss Tecator, Sweden) at 420 °C for 30-40 minutes. After cooling, the mineralized samples were steam distilled in a Kjeltec TM 2200 no. (Foss Tecator, Sweden) with a 4% boric acid solution in the collector. The distillate was titrated with 0.2 M sulfuric acid in the presence of a Tashiro indicator to obtain a pink color.

The protein content (B) was calculated from the formula:

B = (Vx1.75x6.25)/m,

where: V-volume of sulfuric acid used for titration (cm^3), 1.75 - amount of nitrogen, which corresponds to 1 cm 0.2 M sulfuric acid used for titration, 6.25- conversion factor of nitrogen content into protein content in meat raw materials, and m - sample mass (mg).

4) Fat content

Fat content by the Soxhlet method [PN ISO 1444: 2000],

The method consists in determining the free fat content by the Soxhlet method.

A sample of mass 2.5 g was weighed with an accuracy of 0.001 g and dried according to the procedure in ISO 1442 standard. The flask of the extraction apparatus with a few glass beads placed in it was dried for 1 hour in an oven (Oven Series 9000, Thermolyne, United States) at 103 °C. It was cooled down in a desiccator to room temperature and weighed to an accuracy of 0.001 g. The dried weight sample was quantitatively transferred from the weighing plate to the extraction thimble. The remains of the dried weight sample were removed from the plate with cotton wool moistened with solvent, and the cotton wool was also put into the thimble. The thimble was placed in the extraction chamber. The flask was connected to an extraction apparatus (Soxtec System HT 1043 Extraction Unit, Tecator Co., Sweden). Extraction was carried out for 6 hours. After evaporation of the solvent, the flask with fat was dried for 1 hour in a laboratory oven at 103 °C, then cooled in a desiccator to room temperature and weighed with the accuracy of 0.001 g.

The free fat (T) content (in percent) was calculated as follows: T (%) = (m2 - m1) x 100/m0.

where: m0 - mass of the sample before drying, m2- mass of the extraction flask with the fat after drying, m1- mass of the extraction flask without fat.

5) Water content

Water content by drying method [PN ISO 1442: 2000],

The method consists in thoroughly mixing the sample with sand and drying it to constant mass in a laboratory dryer (Oven Series 9000, Thermolyne, United States) at 103 °C.

The weighing plate with sand, with mass three to four times larger than the mass of the sample, and the glass rod, was dried for 30 minutes in a laboratory drier at 103 °C. The sand plate with the glass rod was cooled down to room temperature in a desiccator and weighed with an accuracy of 0.001 g. 5 to 8 g of the sample was transferred to the plate prepared in this way, and then the plate with the contents and glass rod was weighed with an accuracy of 0.001 g. Subsequently, the contents of the plate were mixed well with a glass rod. The plate with its contents and the glass rod were dried in an oven at 103 °C for 2 h, then removed from the oven and placed in a desiccator. Plates were cooled to room temperature and weighed with an accuracy of 0.001 g.

The water content (W) as percentage by mass was calculated using the equation:

$W = (m1-m2)/(m1-m0) \times 100\%$,

where: m0- mass of the plate with sand and a glass rod;

m1-mass of plate with sample, sand and glass rod before drying;

m2- weight of the plate with sample, sand and a glass rod, after drying.

II. Validation methods

The contents of calcium and sodium in chicken meat were analyzed using validated methods. Reference material Matrix Meat Reference Material (SMRD 2000) was used to assess the validation parameters (Table 2). Analytical quality assurance and the technical performance of the analysis used for the determination of calcium and sodium were checked in FAPAS 04309 inter-laboratory studies.

Table S2. Validation parameters.

Parameters	Ca	Na
The limit of quantification LOQ), mg/kg	0.40	0.10
The limit of detection (LOD), mg/kg	0.20	0.05
Recovery, (%)	100.6	99.0
Linearity	0.9998	0.9995
Precision, %	2.1	3.5
